# Curvature‐Enhanced Superomniphobic Property for Minimizing Contact Time of Low‐Surface‐Tension Liquid

**DOI:** 10.1002/smsc.202400631

**Published:** 2025-03-04

**Authors:** Hyunah Ahn, Geun‐Tae Yun, Jin Ryu, Gyu‐Min Jang, Sung Gap Im, Hee‐Tae Jung

**Affiliations:** ^1^ Department of Chemical and Biomolecular Engineering (BK21 four) Korea Advanced Institute of Science and Technology (KAIST) 291 Daehak‐ro Yuseong‐gu Daejeon 34141 Republic of Korea; ^2^ Functional Thin Film Laboratory (FTFL) Department of Chemical and Biomolecular Engineering Korea Advanced Institute of Science and Technology (KAIST) 291 Daehak‐ro Yuseong‐gu Daejeon 34141 Republic of Korea; ^3^ Hydrogen and Low‐Carbon Energy R&D Lab Posco Holdings Pohang 37637 Republic of Korea

**Keywords:** asymmetric bouncing, contact times, hierarchical structures, superomniphobic surfaces, surface curvatures

## Abstract

In nature, the springtail represents an ideal superomniphobic system, exhibiting remarkable resistance to organic liquids in both static and dynamic states. This behavior is attributed to the hierarchical structure of their skin, consists of micro‐ and nanostructures. While numerous artificial superomniphobic surfaces have been developed to mimic its geometry and properties, previous designs are limited to flat surfaces and failed to incorporate the curvature of the springtail's cuticle. Here, a curved superomniphobic surface is first developed that mimics both the curved shape and hierarchical structure of springtail skin. This system developed on the flexible substrate reveals the significant role that curvature plays in reducing the contact time of low‐surface‐tension liquid. While the static repellency on curved and flat surfaces is comparable, droplet rebound dynamics are distinctive on curved surfaces, showing asymmetric bouncing that conforms to the curvature. This effect intensifies with increased curvature, leading to a reduction in contact time by up to 54%, a record for organic liquid. This study uncovers the crucial role of surface curvature in springtail superomniphobicity and offers valuable insights for designing advanced omniphobic systems.

## Graphical abstract

A curved superomniphobic surface inspired by springtail skin reduces a droplet contact time by over 50% for low‐surface‐tension liquids. By replicating both the curvature and hierarchical structures of the springtail skin, droplets rebound asymmetrically, minimizing contact time. This innovation paves the way for designing highly repellent surfaces applicable to various liquids for practical and industrial uses.

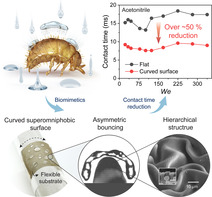

## 1 Introduction

Springtail (*Collembola*) found in nature are considered the most ideal omniphobic creature that exhibiting remarkable static and dynamic repellency to liquids.^[^ ^1^ ^]^ Their cuticle repels oils in a static state (contact angle (CA) >150°) and remains unwetted by impacting droplets such as raindrops, with elevated hydrostatic pressure up to 4200 hPa.^[^ ^2^ ^]^ It is well reported that such robust repellency results from the unique structure of their skin, which consists of a multiscale hierarchical structure incorporating hexagonally arranged nanoscale re‐entrant structures as primary granules and microscale groove as a secondary granule.^[^ ^3^, ^4^, ^5^ ^]^ As the hierarchical surface minimizes the solid–liquid contact area, liquids can be sustained at the top of the skin. The primary and secondary granules could also dissipate the impact pressure, thereby providing robust air pockets that prevent droplet from penetrating the cavities.^[^ ^6^ ^]^ Additionally, from a macroscopic perspective, the skin surface has a curved, particularly convex, geometry. These complex configurations can interact with liquids, resulting in nonwetting properties.

Over the past few years, artificial superomniphobic surfaces mimicking the unique skin structure of the springtail have been proposed, including overhang‐shaped,^[^ ^7^, ^8^ ^]^ doubly re‐entrant structures,^[^ ^9^, ^10^, ^11^ ^]^ and micro‐ and nanostructure combined hierarchical surfaces.^[^ ^12^ ^]^ Overhang‐shaped structures were fabricated on rigid substrates by conventional microelelectromechanical processes, mimicking the nanoscopic comb‐like pattern of the springtail cuticle, which establishes a pressure barrier and inhibits the liquid penetration into the surface grooves.^[^ ^13^ ^]^ Doubly re‐entrant micropillars on a Si substrate, reminiscent of a serif‐T shape where the thin and short wall droops down vertically under the flat head, were reported. As this structure maximizes the liquid‐supportive air pockets up to 95%, any organic liquids (e.g., 10 mN m^−1^ of tetradecafluorohexane (FC‐72)) can roll over this surface without collapsing its own surface tension. Another superomniphobic surface recently reported further enhanced its repellency, demonstrating the complete rebound of ethanol droplet with a Weber number (*We*) ≈104.^[^ ^12^, ^14^ ^]^ As this system reproduces springtail's skin structure more precisely, incorporating doubly re‐entrant nanostructures on microgrooves, robust air pockets are formed, effectively repelling the droplet even under dynamic pressure.

**Figure 1:**
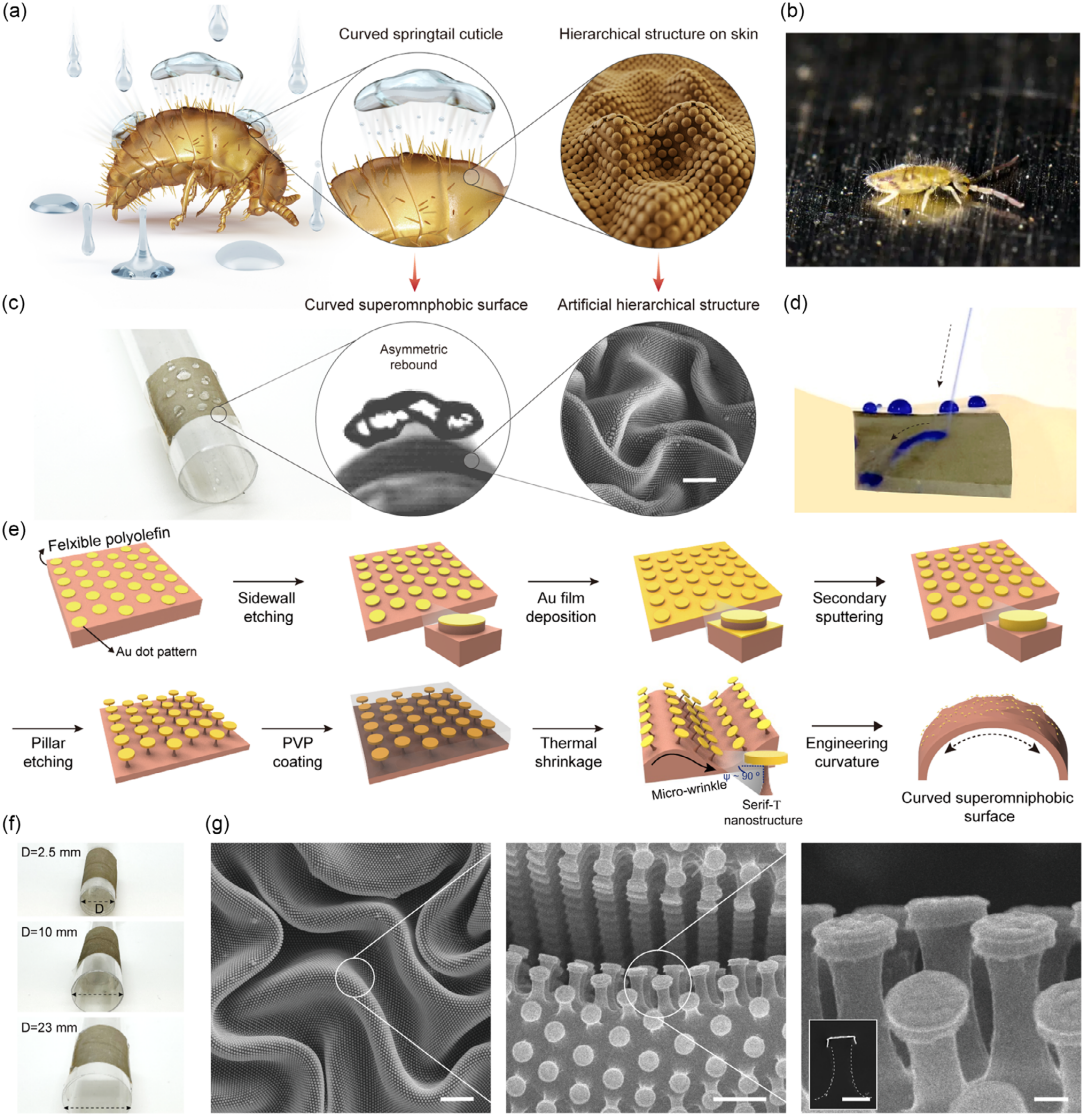
Design of the curved superomniphobic surface inspired by the springtail. a) Schematic of the springtail with curvature and hierarchical structures. b) Photograph images of the springtail resisting raindrops. c) Photograph of the curved superomniphobic surface and SEM image of fabricated hierarchical structures. Scale bar, 10 μm. d) Photograph of curved superomniphobic surface shielding organic liquids. e) Schematic of fabricating the curved superomniphobic surface. f) Photograph of curvature‐controlled surfaces. g) SEM images of the superomniphobic surface with hierarchical structure incorporating microwrinkle and serif‐T‐shaped nanostructures in top‐view and cross‐sectional view (inset). Scale bars: 10, 1 μm, and 200 nm.

While these studies achieved superior nonwetting properties, they are limited to the flat state substrate (e.g., Si wafer, rigid polymeric substrate) as the fabrication technologies to prepare complex hierarchical structure are hard to be adapted on flexible substrates. The springtail cuticle observed in nature exhibits a macroscopically convex morphology with underlying hierarchical structure (**Figure** **1**a).^[^ ^15^ ^]^ Owing to this unique topography of the skin, it remains unwettable by aqueous and organic liquid droplets (Figure 1b). This implies that macroscopic curvature could potentially influence nonwetting properties in the design of superomniphobic systems. Additionally, the role of surface morphology in water droplet behavior was extensively explored for practical applications and found to advance dynamic outcome.^[^ ^16^, ^17^, ^18^, ^19^, ^20^, ^21^ ^]^ The interweaving effects of omniphobic behavior and surface macroscopic curvature have not been addressed so far. While some flexible substrates (e.g., polymers, polydimethylsiloxane (PDMS)) was utilized for the fabrication of superomniphobic surface by the nanoimprint soft molding methods, these surfaces are not capable of repelling impacting liquids, which obscures the influence of surface curvature on dynamic repellency.^[^ ^10^, ^22^, ^23^ ^]^ Due to these limitations, the interweaving effects of surface‐curved geometry and its impact on omniphobic properties have yet to be investigated.

To address these challenges, we fabricated a curved superomniphobic surface inspired by the springtail's convex form and demonstrated the role of macroscopic curvature of the surface in advancing superomniphobic properties. Our superomniphobic system was fabricated on a flexible substrate that allows for surface curvature modulation, incorporating a springtail mimetic hierarchical structure that builds upon a previously developed design using nanolithography and thermal wrinkling processes (Figure 1c). We observed that the static repellency to low‐surface‐tension liquids (CA > 150°) remained comparable to flat surface, while the droplet dynamics were distinguished from the flat states showing asymmetric rebounds shielding the surface from wetting even upon harsh impacts (*We*: ≈400) (Figure 1d). The effect of surface curvature in acetonitrile droplet dynamics were evaluated by controlling its curvature, which demonstrated that asymmetry during spreading and retraction became more pronounced as the curvature increased. Remarkably, preferential fluid retraction induces rapid droplet rebounds with 56% reduced contact time compared to the flat surface. This reduction is significant for low‐surface‐tension liquids, which have been less explored in dynamic repellency studies (Table S1, Supporting Information). We validated significant role of macroscopic curvature in improving omniphobic properties, providing valuable insights into the precise design of liquid repellent surfaces and their implementation in practical applications.

## 2 Results

### 2.1 Design of Flexible, Curved Superomniphobic Surface with Springtail Mimetic Hierarchical Structure

To investigate the unique role of curvature in omniphobic properties, we developed curved superomniphobic surface that mimics the multiscale structure of springtail skin from the macro‐ to nanoscale. A flexible polyolefin (PO) substrate was utilized to fabricate the hierarchical structure, offering greater flexibility and mechanical durability while maintaining the ability to form well‐defined microwrinkles.^[^ ^24^, ^25^ ^]^ First, the Au‐dot pattern fabricated on the SiO_2_ substrate was transferred onto a PO substrate by using the polymethylmethacrylate (PMMA)‐assisted method (Figure S1, Supporting Information). Using this dot pattern as an etching mask, the side wall was etched by reactive ion etching (RIE), and a 40 nm‐thick Au film was deposited by e‐beam evaporation. Through the secondary sputtering phenomenon, Au was sputtered on the sidewall, and the pillar was etched by RIE.^[^ ^26^, ^27^ ^]^ Then, the doubly re‐entrant serif‐T (T) shaped nanostructure with a 90° of geometrical edge angle was fabricated. Second, microwrinkles were continuously generated by a heat‐induced thermal shrinkage method, where the applied areal strain (*ε* = (A–A_0_)/A_0_, where A and A_0_ represent projected areas before and after strain relief, respectively) was fixed as 0.75.^[^ ^28^, ^29^ ^]^ This was followed by initiated chemical vapor deposition (iCVD) for pHFDMA coating.^[^ ^30^ ^]^ The multiscale hierarchical structure was finally created over a large area (≈3 × 3 cm^2^) with high uniformity, possessing consistent superomniphobic properties across the entire area (Figure S2, Supporting Information). Finally, the fabricated substrate was rolled to engineer the curvature of our system, and the curved superomniphobic surface was fabricated.

Furthermore, the surface curvature can be widely adjusted from a steep curvature comparable to the droplet size to gentle one close to flat (curvature diameter: ≈2.5 to ≈23 mm), which is useful to investigate curvature‐dependent wetting behaviors and adaptable to curved surfaces (Figure 1f). The scanning electron microscopy (SEM) images represent the hierarchical structure consisting of a uniform arrangement of hexagonal arrays of serif‐T‐shaped nanostructures (D: 450 nm, h: ≈710 nm) positioned on periodic microwrinkles (λ, 10.32 μm; A, 9.92 μm) across the entire area (Figure 1g). This specific geometry provides air pockets at micro‐ and nanoscales essential for extreme repellency. Our biomimetic approach offers the advantage of precise, independent modulation of each structural component, including curvature and the dimensions of nano‐ and microstructure dimensions. The microwrinkle wavelength (λ: 5.08–16.52 μm) and amplitude (A: 5.75–10.64 μm) were adjusted by controlling skin layer thickness during thermal shrinkage (Figure S3a, Supporting Information). Additionally, nanostructure head diameter (D: 200–650 nm) and pillar height (h: ≈800 nm) were tailored by tuning RIE‐etching parameters (Figure S3b, Supporting Information).

### 2.2 Static Repellency on the Flat and the Curved Artificial Springtail Surfaces

**Figure 2:**
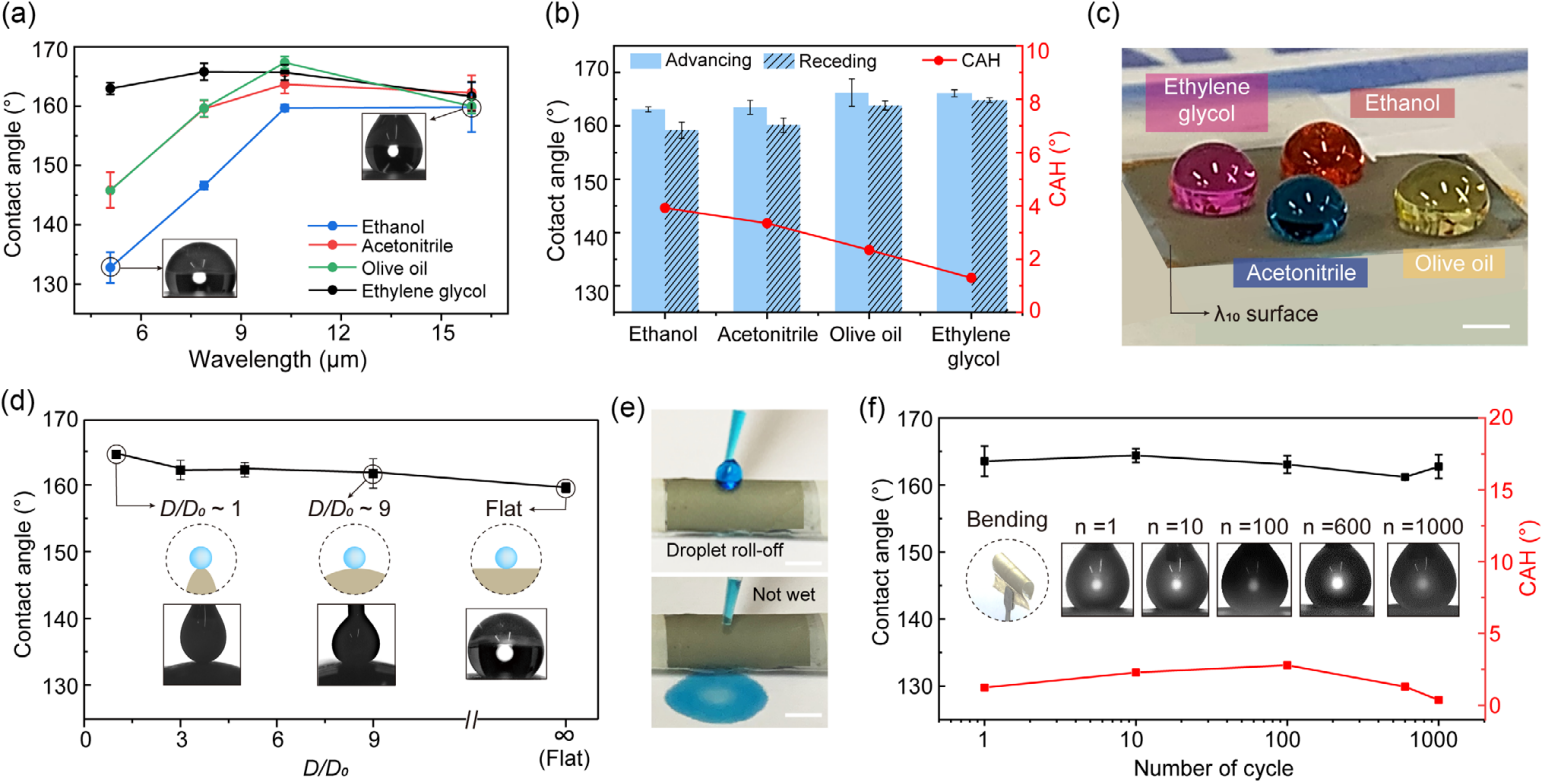
Static repellency of various organic liquids on flat and curved superomniphobic surfaces. a) Apparent contact angles of various organic liquids (ethanol, acetonitrile, olive oil, and ethylene glycol) on flat surfaces with four different wrinkle dimensions (λ_5_, λ_8_, λ_10_, and λ_17_). b) Dynamic contact angles and CAH of tested organic liquids on flat λ_10_. c) Photograph image of dyed organic liquids on flat λ_10_. Scale bar, 2 mm. d) CA of ethanol on surface curvature engineered surfaces (*D/D*_0_ ≈ 1, 3, 5, and 9). e) Ethanol droplet rolling off from *D/D*_0_ ≈ 1 surface. f) Bending stability of superomniphobic property under 1000 cycles of bending (*D/D*_0_ ≈ 1). Error bars denote the standard deviation over three experiments. Data are presented as the mean ± standard deviation (*n* = 3).

**Figure** **2** shows the static repellency of various liquids on the flat and curved artificial springtail surfaces. The omniphobic performance is known to be dependent on the feature shape and sizes of the hierarchical structure.^[^ ^14^ ^]^ According to the Cassie–Baxter relationship, microstructures play a crucial role in enhancing repellency by creating microscale air pockets that prevent droplet penetration, while nanoscale air pockets formed by serif‐T nanostructures are essential for achieving omniphobicity.^[^ ^31^ ^]^ Previous studies have optimized the feature sizes of the serif‐T nanostructures to match those of the springtail, achieving static repellency surpassing that of the springtail. Based on these specifications, we only controlled the microwrinkles generated on PO substrate and optimized their parameters to achieve superior static repellency. By modulating the wavelength (λ), we fabricated four different surfaces (i–iv) as shown in Figure S3a in Supporting Information. From here, we designated each surface based on its wavelength, using the designation “λ_wavelength (μm)_”, such as λ_5_, λ_8_, λ_10_, and λ_17_. We measured the apparent CA (θ*) of representative organic liquids, such as ethanol (*γ* = 22.1 mN m^−1^), acetonitrile (*γ* = 29.29 mN m^−1^), olive oil (*γ* = 32 mN m^−1^), and ethylene glycol (*γ* = 47.7 mN m^−1^) droplets (Figure 2a and Figure S4, Supporting Information).

We observed that the wavelength had a significant impact on static repellency. The surface with a larger wavelength demonstrated stable resilience, effectively resisting the droplet. The λ_10_ and λ_17_ surfaces showed the *θ* ^* ^≈ 160° for all tested liquids, whereas the λ_5_ and λ_8_ surfaces shifted to a Wenzel state for ethanol, acetonitrile, and olive oil with *θ* ^*^ < 160°. In addition, we analyzed the dynamic CA and CA hysteresis (CAH, CAH = Advancing CA – Receding CA) of the four organic liquids on each surface (Figure 2b and Figure S5a–g, Supporting Information). All liquid droplets rolled off smoothly (CAH < 4°) from λ_10_ and λ_17_, and moreover, the droplets were not even deposited on the surface, indicating a significantly low‐surface energy of these surfaces (Figure S5h, Supporting Information). However, CAH gradually increased on smaller wavelength surfaces (λ_5_ and λ_8_) up to 15°, where the droplets were pinned in a Wenzel state. Other organic liquid droplets with varying viscosities, densities, and surface tensions also demonstrated superior repellencies (*θ* ^*^ ≈ 160° and CAH < 5°) on the λ_10_ surface (Figure S6a and Table S2, Supporting Information). We also observed the positioning of droplets on the λ_10_ surface: ethanol (red), acetonitrile (blue), olive oil (yellow), and ethylene glycol (pink) (Figure 2c). The strong repellency on the λ_10_ and λ_17_ surfaces can be attributed to the smaller contact fraction between the droplet and microscale wrinkles. The larger microscale cavities provide a significantly smaller contact area but form robust air pockets capable of repelling low‐surface‐tension liquid consistent with the Cassie–Baxter relationship. Therefore, we concluded that the wrinkle wavelengths around 10–17 μm were beneficial for suspending organic liquids.

We further evaluated the static repellency of the curved superomniphobic surface, which was created by rolling a flat substrate to achieve different curvatures. The curvature of curved surface was modulated into four different types, with each surface named based on its *D*/*D*_0_ ratio—≈1, 3, 5, and 9—where the diameter of curvature (*D*) is normalized to the initial droplet diameter (*D*_0_). Ethanol was dropped as a representative liquid, and *θ* ^*^ was measured on each surface by pinning the droplet on the surface to prevent roll‐off (Figure 2d). Analogous to the flat surface (*θ* ^*^ ≈ 160°), all the curved surfaces exhibited contact angles exceeding 160°, and the droplets were not even deposited. This suggests that static repellency can be preserved even when altering the curvature of a superomniphobic surface. Additionally, we proceeded to measure the static repellency under a repeated bending test (bent the surface *D/D*_0_ < 1). When ethanol was dropped on the *D/D*_0_ ≈ 1 surface, the droplet easily rolled off without wetting the surface, even after repeated tests (Figure 2e). Other low‐surface‐tension liquid droplets with varying fluidic properties also slide off from the surface, leaving unwetted (Figure S6b, Supporting Information). Figure 2f demonstrates that the nonwetting performance was maintained (*θ* ^*^ ≈ 160°, CAH: ≈3°) even after 1000 cycles of repetitive bending and releasing. The air pockets created by the hierarchical structure remained intact, not ruptured even with macroscopic deformation over an extended cycle. The surface also demonstrated thermostability, maintaining *θ* ^*^ above 150° and CAH below 5° across a temperature change ranging from room temperature to 200 °C (Figure S7a,b, Supporting Information). This stability was attributed to the durable hierarchical structure, which remained intact despite temperature fluctuations. To further evaluate its chemical resilience, the surface was immersed in strong acid (pH 1), saline (pH 7.4), and strong alkali (pH 14), followed by CA measurements (Figure S7c, Supporting Information). The surface retained is static repellency (*θ* ^*^ > 155°, CAH < 5°), indicating robust resistance to extreme pH conditions. These results confirm that our superomniphobic surface possesses exceptional bending, thermal, and chemical robustness, reinforcing its suitability for practical and industrial implementation.

Therefore, we found that the curved superomniphobic surface with optimized microwrinkle morphologies has excellent performance in static repellency, comparable to that of the flat surface.

### 2.3 Asymmetric Bouncing on the Curved Superomniphobic Surface with Reduced Contact Time

**Figure 3:**
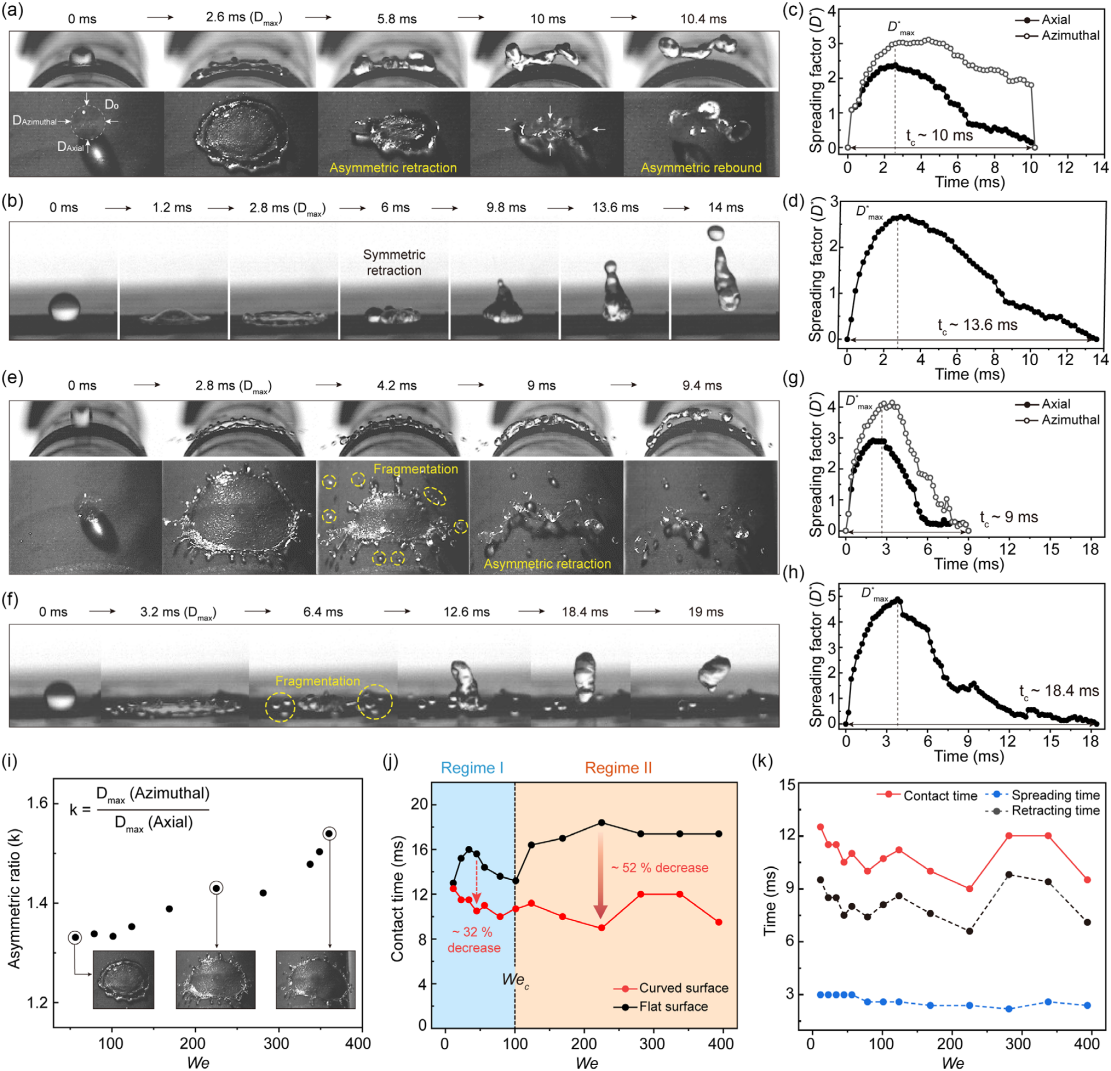
Asymmetric bouncing of acetonitrile droplet on the curved surface (*D/D*_0_ ≈ 5) which induces rapid droplet rebound. a,b) Sequential snapshot images (top and side view) of (a) asymmetric bouncing at *We* ≈79 on the curved surface (*D/D*_0_ ≈ 5) and (b) droplet rebound at *We* ≈79 on the flat surface. c,d) Temporal evolution of dimensionless contact line distance (*D* ^*^) in the axial and azimuthal directions at *We* ≈79 on the (c) curved surface and (d) flat surface. e,f) Sequential snapshot images (top and side view) of asymmetric bouncing (e) at *We* ≈225 on the curved surface (*D*/*D*_0_ ≈ 5) and droplet rebound (f) at *We* ≈225 on flat surface. g,h) Temporal evolution of dimensionless contact line distance (*D* ^*^) in the axial and azimuthal directions at *We* ≈225 (g) on the curved surface and (h) on the flat surface. i) The spreading asymmetry ratio (k) under different *We.* j) Contact time on the curved surface compared to the flat surface under different *We*. Regime I, asymmetric bouncing; Regime II, asymmetric bouncing with splash. k) Spreading and retracting time constituting contact time under different *We*.

Based on the advanced static repellency of our curved superomniphobic system, we proceeded to evaluate its dynamic repellency by monitoring the droplet impact and rebound dynamics (**Figure** **3**). We used a high‐speed camera at up to 5000 frames per second to precisely capture the behaviors and characterized the phenomenon by the *We* = $\frac{\rho D_{0}V_{0}^{2}}{\gamma }$, which is the ratio of inertial force to capillary force; *ρ*, *γ, V*_0_, and *D*_0_ represent the liquid density, surface tension, initial droplet velocity, and initial droplet diameter, respectively. Among the λ_10_ and λ_17_ surfaces showing a large CA for acetonitrile, the λ_10_ surface was investigated as it resulted in the complete rebound of various organic liquids over a wide *We* range (Figure S8, Supporting Information). We selected acetonitrile for testing as it allows a general investigation of low‐surface‐tension liquid repellency considering its surface tension (*γ*: 29.29 mN m^−1^) and its common usage in various applications.^[^ ^32^ ^]^ The acetonitrile (*D*_0_: 2.14 mm) was dropped onto the curved (*D/D*_0_ ≈ 5) and flat surfaces with strong impact energy (*We* up to 400).

Figure 3a,b displays the sequential droplet rebound images at We ≈79 on the curved and flat surfaces, respectively (Video S1, Supporting Information). The droplet spread and retracted anisotropically on the curved surface, in contrast to the symmetric behavior observed on the flat surface. The temporal evolution of the spreading factor (D*) in the axial and azimuthal directions clearly exhibited the asymmetrical phenomena on the curved surface, where *D* ^*^_max_ reached to ≈3.1 in the azimuthal direction but only ≈2.4 in the axial direction (*t* = 2.5 ms). Furthermore, it retracted fully in the axial direction, while only partially in the azimuthal direction (*t* = 10 ms), leading to an elongated shape and asymmetric bouncing. From side view images, we observed a significant increase in the rim size in the azimuthal direction, and the droplet's center was pushed upward due to macrotexture‐induced buoyancy effects.^[^ ^33^ ^]^ Meanwhile, the droplet spread symmetrically on the flat surface reaching to D* ≈ 2.8 at maximum spreading and retracting to a circular droplet form (*t* = 13.6 ms), as shown in Figure 3d. The contact time, the duration for which a droplet remains in contact with the surface before it completely rebounds, is shortened on the curved surface compared to the flat surface from 13.6 to 10 ms.

Next, we analyzed rebound dynamics under more intense conditions (*We*: ≈225) and likewise observed the asymmetric behaviors during spreading and retraction on the curved surface (Figure 3e,f and Video S2, Supporting Information). We also observed droplet fragmentation ejecting some small‐sized secondary droplets, caused by the instability at the interface between air pockets from the rough surface and spreading liquids.^[^ ^34^, ^35^, ^36^ ^]^ The drop spread extensively reaching to *D* ^*^_max_ ≈ 4 in the azimuthal direction (*t* = 2.8 ms) due to high impact inertia, and notably, the droplet on the curved surface rebounded much more rapidly (*t* = 9 ms) compared to the flat surface (*t* = 18.4 ms) (Figure 3g,h). This unusual phenomenon can be attributed to the synergistic effects of asymmetric rebound and droplet fragmentation, which reduces the effective volume of each droplet to bounce‐off the surface. Therefore, the fragmented droplets on the curved surface rebounded remarkably with 52% reduced contact time.

To quantify the asymmetric behaviors on the curved surfaces, we first measured the asymmetric ratio (k), defined as the ratio of diameter in the axial (*D*_axial_) and azimuthal direction (*D*_azimuthal_) in the range of 0 < *We* < 400 (Figure 3i). The ratio k increased with elevating *We* because of greater spreading inertia with higher energy impact. According to the mechanism demonstrated by the lattice Boltzman simulation, momentum is preferentially transferred to the short axis on the elliptical impact area, causing the fluid to flow preferentially in the azimuthal direction.^[^ ^37^, ^38^, ^39^ ^]^ Furthermore, the contact time (*t*_c_) across the entire *We* range was shortened on the curved surface compared to the flat surface because of asymmetric bouncing, indicating that the affinity between the liquid and the surface was weaker on the curved surface (Figure 3j). We categorized the *We* range into two regimes based on a critical *We* (*We*_c_), which marks the onset of droplet splash when inertia overcomes capillary force. In regime I (*We* <*We*_c_, *We*_c_ ≈ 101), *t*_c_ was reduced by up to 32% (*We*: ≈45) as shown in Figure 3a (*We*: ≈79). Regime II (*We*_c_ < *We* < 400) encompasses the range where droplet splash and fragmentation appear as illustrated in Figure 3b. In this regime, *t*_c_ was significantly shortened by as much as 52% (*We*: ≈225), a remarkable achievement for organic liquid. While some studies have reported reducing the contact time of water droplets on hydrophobic surfaces based on various approaches, the studies on organic liquids achieving shortened contact time were impeded due to limited surface repellency.^[^ ^34^, ^36^, ^37^, ^38^ ^]^ We also found that the contact time reduction is caused by asymmetric retraction behaviors as the retracting time dominates *t*_c_ compared to the spreading time (Figure 3k).

### 2.4 Surface Curvature‐Dependent Asymmetric Bouncing Dynamics

**Figure 4:**
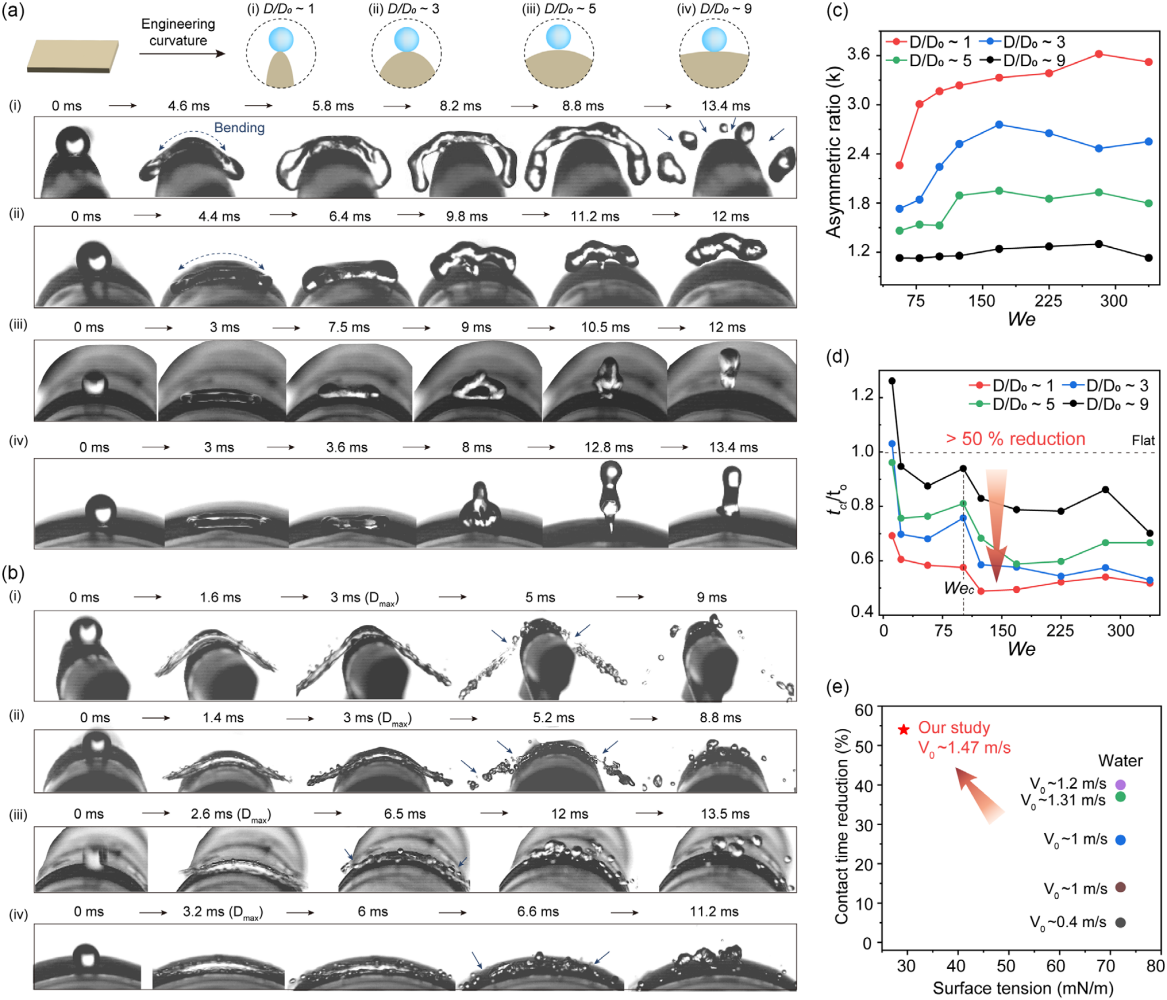
Evaluation of asymmetric bouncing on curvature‐engineered surfaces (*D/D*_0_ ≈ 1, *D/D*_0_ ≈ 3, *D/D*_0_ ≈ 5, *D/D*_0_ ≈ 9). a,b) Sequential snapshot images of acetonitrile asymmetric bouncing behaviors on curvature‐engineered surfaces (a) at *We* ≈45 and (b) at *We* ≈337.5. c) The asymmetric ratio (k) as a function of *We* on each curved surface. d) Contact time reduction as a function of *We* on each curved surface compared to the flat surface. e) Comparison of contact time reduction (%) between our study with previous literature.

To evaluate the surface curvature dependency on asymmetric bouncing behaviors, the curvature of our surface, λ_10_, was modulated to achieve *D/D*_0_ ratios of ≈1, 3, 5, and 9. We then dispensed acetonitrile onto each surface with a *We* ranging from 0 to 350, covering strong impact conditions previously unexamined. Various droplet dynamics, including spreading, retracting, and splashing, were observed using a high‐speed camera. Initially, we focused on the droplet with gentle impacts (e.g., *We*: ≈45) on each curvature (**Figure** **4**a and Video S3, Supporting Information). The droplet spread by bending along the surface curvature particularly on higher curvature surfaces such as *D/D*_0_ ≈ 1 and *D/D*_0_ ≈ 3, whereas the droplet spread without bending on *D/D*_0_ ≈ 5 and *D/D*_0_ ≈ 9. Likewise, the retraction was more asymmetric as the surface curvature increased. On the *D/D*_0_ ≈ 1 surface, the droplet retracted primarily in the axial direction and fragmented droplets were split off even without converging (*t* = 8.2 ms). Furthermore, we investigated droplet behaviors under severe impact conditions with extreme pressure (e.g., *We*: ≈337.5) (Figure 4b and Video S4, Supporting Information). Compared to the gentle impact cases, the intense impact inertia caused the droplets to spread preferentially in the azimuthal direction with droplet fragmentation ejecting secondary droplets. After reaching maximum spread, the liquid film ruptured and did not converge on all curvatures (indicated by arrows in Figure 4b) as observed on the *D/D*_0_ ≈ 1 surface at *We* ≈45 (Figure S9, Supporting Information). This converging breakup tends to be initiated under strong impact condition with higher *We* and on surfaces with higher curvature and low *D/D*_0_ surface (Figure S10, Supporting Information).^[^ ^40^ ^]^ As the droplet stretched beyond the supporting solid surface, the rim ends unsupported by the surface was broken‐off, and fragmented droplets in the center ultimately rebounded, not wetting the surface.

To quantify the effect of surface curvature on asymmetric behavior, we plotted the coefficient k and splashing angle (*θ*) as a function of *We* (Figure 4c and Figure S11, Supporting Information). The surfaces with higher curvature significantly amplified the coefficient k, indicating an increase in behavioral asymmetry. The angle *θ* defined as the intersection angle formed by the spreading lamella along the curve was reduced with increasing surface curvature. We also evaluated the contact time reduction (*t*_*c*_ */t*_0_, *t*_0_ is the contact time on a flat surface) and observed that the reduction was more pronounced on curvature amplified surface (Figure 4d), implying that surface curvature facilitates a rapid rebound induced by asymmetric retraction (Figure S12, Supporting Information).^[^ ^38^ ^]^ The contact time was more evidently reduced by over 50% as *We* increased beyond *We*_c_ due to additional fragmentation effects, with a notable reduction of 54% on *D/D*_0_ ≈ 1 at *We* ≈125. Considering that the research on reducing contact time for organic liquids has been hindered by limited surface repellency, our accomplishment is noticeable for low‐surface‐tension liquid with fast impact velocity (Figure 4e and Table S1, Supporting Information).^[^ ^18^, ^38^, ^41^, ^42^, ^43^, ^44^ ^]^

Beyond the enhanced bouncing behaviors, the self‐cleaning capability of the surfaces was also evaluated by introducing contaminant particles onto the substrate and observing their removal under droplet impact. The curved surfaces facilitated efficient self‐cleaning, as the asymmetric retraction of droplets generated strong directional forces, effectively detaching contaminants from the surface (Figure S13, Supporting Information).

## 3 Conclusion

In this study, we demonstrated the crucial role of surface curvature in superomniphobic properties by reducing the contact time of low‐surface‐tension liquid. The surface developed on a flexible substrate, inspired by the curved skin morphology of the springtail and the hierarchical structure of its cuticle, enables the investigation of how the surface curvature influences static and dynamic repellency. We identified that the static repellency on the curved surface was consistent with that on the flat surface, whereas the droplets colliding on the curved surface exhibited distinctive asymmetric bouncing. These unusual dynamics became more pronounced with increasing surface curvature, enabling a remarkable contact time reduction by up to ≈54% for acetonitrile. Through this validation, we propose that the curved shape of springtail skin contributes to its extreme repellency. The role of macroscopic curvature on superomniphobic surface as well as fabrication technology of hierarchical structure onto the flexible substrate would be emphasized to advance the springtail mimetic superomniphobic surface. Future directions include fluid simulations of asymmetric dynamics with fluidic properties for precise analysis supporting our results. Additionally, future studies expand the range of organic liquids and curvature types including concave or complicated shape. We also believe that this insight could be valuable for designing practical applications requiring superomniphobic properties.

## 4 Experimental Section

4.1

4.1.1

##### Fabrication of Springtail Inspired Hierarchical Structure

The springtail‐inspired hierarchical structure was fabricated in two steps: fabrication of serif‐T‐shaped nanostructure and formation of microscale wrinkle. First, an Au‐nanodot pattern was prepared by the following procedures. A PDMS mold (Sylgard 184, 10:1 (weight ratio) prepolymer/curing agent; Dow Corning) was replicated from a silicon hole pattern (depth, 350 nm; pitch, 1 μm; diameter, 500 nm). A thin polystyrene (PS) film was spin‐coated onto a 40 nm thick Au film deposited on a silicon–oxide substrate. The PDMS mold was pressed onto the PS‐coated surface, and the PS filled the voids of the mold by capillary force heated above the glass transition temperature (*T*_g_ = 120 °C). Then, the polymer prepattern was etched by RIE O_2_ plasma, and the exposed Au layer was etched by Ar^+^ ion milling. The remaining PS cylinder was etched by RIE O_2_ plasma, and the final gold dot pattern with a diameter of 500 nm was transferred onto the shrinkable PO by the PMMA‐assisted method. The edge angle (*ψ*) ≈90° of the serif‐T‐shaped nanostructures was fabricated by RIE O_2_ plasma etching at high vacuum condition (3.0 × 10^−6^ Torr). On the surface, a 30 nm‐thick Au film was deposited by e‐beam evaporation. The Au film was etched by low‐energy Ar^+^ gas, and the bottom gold layers were resputtered onto the sidewall of the PS pattern via a secondary sputtering phenomenon. Then, the pillar was etched by RIE O_2_ plasma, and the serif‐T‐shaped nanostructure was finally formed.

The microscale wrinkle was continuously generated over a large area by a heat‐induced thermal shrinkage process. On the PO sheet with the serif‐T‐shaped nanostructure, polyvinylpyrrolidone (PVP, *M*_w_ = 360 000 g mol^−1^, *T*_g_ = 150 °C) dissolved in ethanol was spin‐coated as a stiff skin layer. Then, the substrate was heated above the glass transition temperature (*T*_g_ = 70 °C) in the oven and shrunk up to an areal strain *ε* (*ε* = (*A*_0_–*A*_f_)/A_0_, where *A*_0_ and *A*_f_ represent projected areas before and after strain relief, respectively) of 0.7. After rinsing the PVP layer, the hierarchical structure was finally fabricated.

##### Modulating the Microscale Wrinkle Morphology

The wavelength and amplitude of the microscale wrinkle were determined by the skin layer thickness during the heat‐induced shrinkage process, which was tuned by the concentration of PVP (*C*_PVP_) dissolved in ethanol. *C*_PVP_ of 5, 7, 10, and 11.5 wt% were used to form λ_5_, λ_8_, λ_10_, and λ_17_ surfaces, respectively. The applied areal strain ε was fixed at 0.75.

##### Surface Curvature Control

The curvature of the substrate was adjusted to *D*/*D*_0_ ≈ 3, 5, 7, and 10 (*D* is diameter of the curvature, and *D*_0_ is the droplet diameter) by rolling the flat substrate along a support material with each curvature.

##### Surface Coating by iCVD

A 3,3,4,4,5,5,6,6,7,7,8,8,9,9,10,10,10‐heptadecafluorodecyl methacrylate (PFDMA, Shanghai Zhuoqin Biotech) and tert‐butyl peroxide (TBPO, Aldrich) were purchased and used as received without further purification. Superhydrophobic polymer pPFDMA was directly synthesized on the structured surface via iCVD (Daeki Hi‐Tech Co. Ltd). PFDMA and TBPO were heated to 75 and 30 °C, respectively. The monomer PFDMA and initiator TBPO were vaporized and introduced into the iCVD chamber. The substrate temperature and the iCVD reactor pressure were kept at 38 °C and 80 mTorr. The flow rate of PFDMA and TBPO was 1 sccm each. The filament temperature was maintained at 140 °C during iCVD operation. The thickness of the polymer was checked in situ by an interferometer.

##### Morphology Characterization

The morphologies of the hierarchical structure were characterized using a Magellan 400 SEM (FEI Company), with an incident energy of the electron beam between 1 and 10 kV. Also, the cross‐section images were observed by a Helios G5 microscope (Thermo Fisher Scientific).

##### CA Measurement

The CA system (Attention, KSV Instruments) was utilized to measure the apparent contact angle. We used ethanol, acetonitrile, olive oil, and ethylene glycol as test liquids. A 6 μL droplet was gently deposited on the omniphobic surface. The advancing and receding contact angles were measured by the same system. The droplets were deposited by a syringe (Hamilton, threaded plunger syringe). The CA was calculated by the OneAttention program. All measurements were performed at room temperature.

##### Temperature and Chemical Stability Tests

For the thermostability test, the superomniphobic surface was incubated on the hot plate at a specific temperature for 30 min. To assess the chemical stability, the surfaces were separately immersed in 0.1 m HCl (pH 1), 1 × PBS (pH 7.4), or 0.1 M KOH (pH 14) for 1 h at room temperature. The surfaces were removed from the solution, rinsed with water, and air‐dried at room temperature. After this treatment, the CA and CAH of the acetonitrile droplet were measured.

##### Droplet Impact Test

The droplet dynamics were recorded by a high‐speed camera (Photron Mini UX50) at 5000 frames/s. Using the syringe pump (Chemyx Fusion 100), the droplets of test liquids (ethanol, acetonitrile, olive oil, ethylene glycol) were released on the surface with constant speed from different heights to obtain various *We*. The height of the droplet was determined by the distance from the tip of the syringe to on top of the curved or flat surface, which was controlled from 1 to 40 cm. The drop impact pressure and *We* were determined by this drop height. The contact time is determined by measuring the duration for which a droplet of liquid remains in contact with the surface before it rebounds. For cases involving droplet fragmentation, the contact time was measured based on the final detachment of the fragmented droplet.

##### Self‐Cleaning Test

The graphite powder as a contaminant was put onto the curved superomniphobic surface. The acetonitrile was then dropped onto the surface to test whether it could remove the contaminant powder, which resulted in a clear and non‐wetted surface.

## Conflict of Interest

The authors declare no conflict of interest.

## Author Contributions

**Hyunah Ahn**: conceptualization (lead); formal analysis (lead); investigation (lead); methodology (lead); visualization (lead); writing—original draft (lead). **Geun‐Tae Yun**: conceptualization (equal); supervision (equal); writing—review and editing (equal). **Jin Ryu**: methodology (equal).**Gyu‐Min Jang**: methodology (equal). **Sung Gap Im**: methodology (equal). **Hyunah Ahn** and **Geun‐Tae Yun** contributed equally to this work.

## Supporting information

Supplementary Material

## Data Availability

The data that support the findings of this study are available from the corresponding author upon reasonable request.
