# Supplementary material for: Curvature‐Enhanced Superomniphobic Property for Minimizing Contact Time of Low‐Surface‐Tension Liquid
Source: Small Sci. 2025 Mar 4;5(6):2400631. doi: 10.1002/smsc.202400631 (PMC12168612; doi:10.1002/smsc.202400631)
Supplement: Supplementary file 1 — Supplementary Material [file SMSC-5-2400631-s001.zip › smsc.12714-sup-0001-suppdata-S1.pdf]

Supplementary Information

Curvature-Enhanced Superomniphobic Property for Minimizing Contact Time of Low-Surface-Tension Liquid

Hyunah Ahn [†], *Geun-Tae Yun* [†], *Jin Ryu, Gyu-Min Jang, Sung Gap Im and Hee-Tae Jung* [*]

H. Ahn, Dr. G.-T. Yun, Prof. H.-T. Jung

Department of Chemical and Biomolecular Engineering (BK21 four), Korea Advanced Institute of Science and Technology (KAIST), 291 Daehak-ro, Yuseong-gu, Daejeon 34141, Korea

E-mail: heetae@kaist.ac.kr

J. Ryu, Prof. S. G. Im

Functional Thin Film Laboratory (FTFL), Department of Chemical and Biomolecular Engineering (BK-21 plus), Korea Advanced Institute of Science and Technology (KAIST), Daejeon 34141, South Korea

Dr. G.-M. Jang

Hydrogen and Low-Carbon Energy R&D Lab, Posco Holdings, Pohang 37637, South Korea

H. Ahn and Dr. G.-T. Yun contributed equally to this work

Keywords: Superomniphobic surface, Curvature, Springtail, Hierarchical structure, Asymmetric bouncing, Contact time, Biomimetic approaches

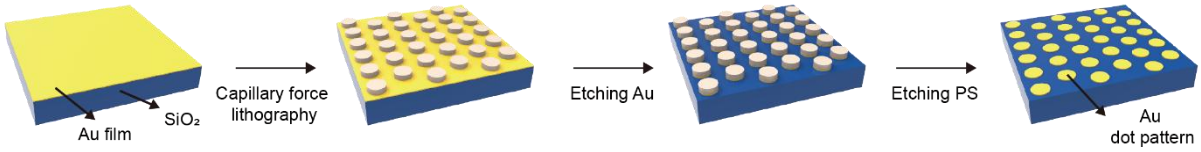

Figure S1. Schematic illustration of fabricating the gold dot pattern used as an etching mask for the serif-T shaped nanostructure.

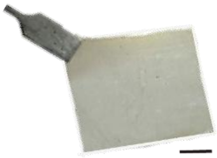

Figure S2. Photograph of the hierarchical structure fabricated on flexible substrate. Scale bar, 1 cm.

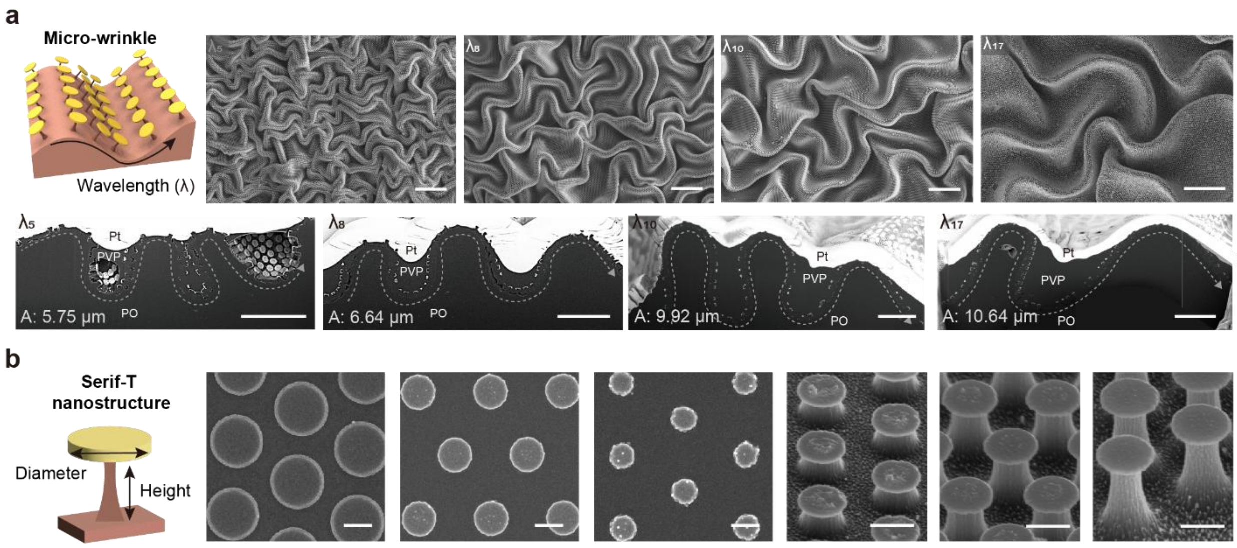

Figure S3. Optimization of dimensions for microwrinkle and serif-T shaped nanostructure that constitute the hierarchical structure. **(a)** SEM images of microwrinkle wavelength (λ) and amplitude-controlled hierarchical structures. Scale bars, 20 μm (Top-view) and 5 μm (FIB sectioned view). **(b)** SEM images of serif-T head diameter and pillar height-controlled structures. Scale bars, 500 nm.

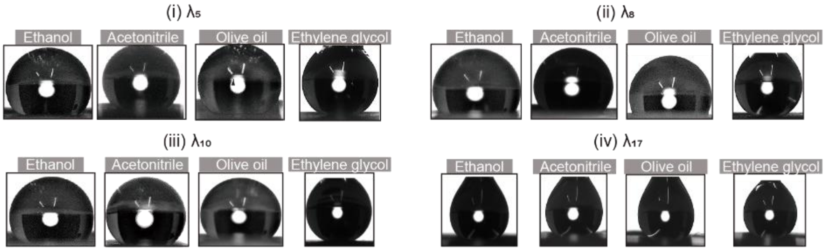

Figure S4. Snapshot images of apparent contact angles on (i) λ_5, (ii) λ_8, (iii) λ_{10}, and (iv) λ_{17} surfaces.

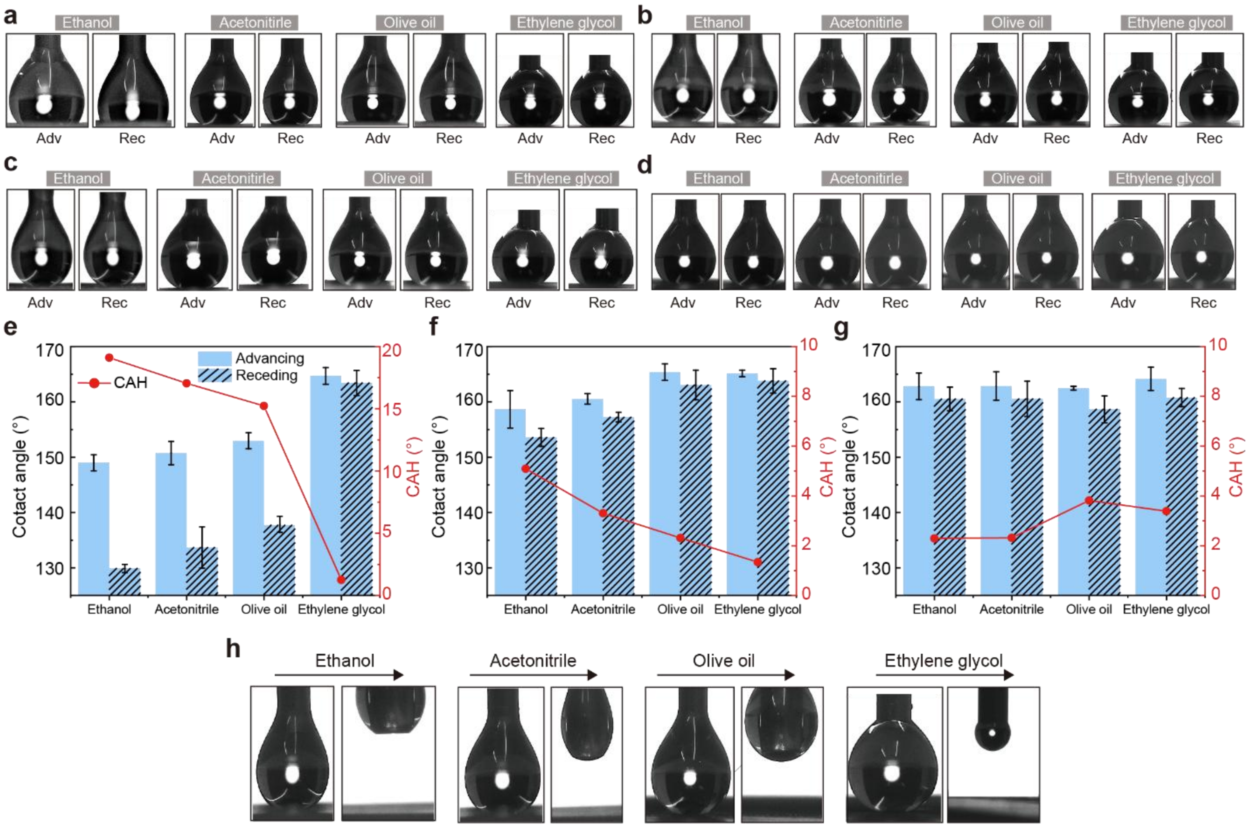

Figure S5. Static repellency of organic liquids on microwrinkle size-controlled surfaces. **(a-d)** Snapshot images of advancing contact angle and receding contact angle of tested organic liquids on (**a**) λ_5, (**b**) λ_8, (**c**) λ_{10}, and (**d**) λ_{17}. **(e-g)** Contact angle hysteresis on surfaces of (**e**) λ_5, (**f**) λ_8, and (**g**) λ_{17}. Data are presented as the mean ± standard deviation (n=3). (**h**) Snapshot images of non-adherent organic liquids on the surface of $\lambda \sim 10$ μm.

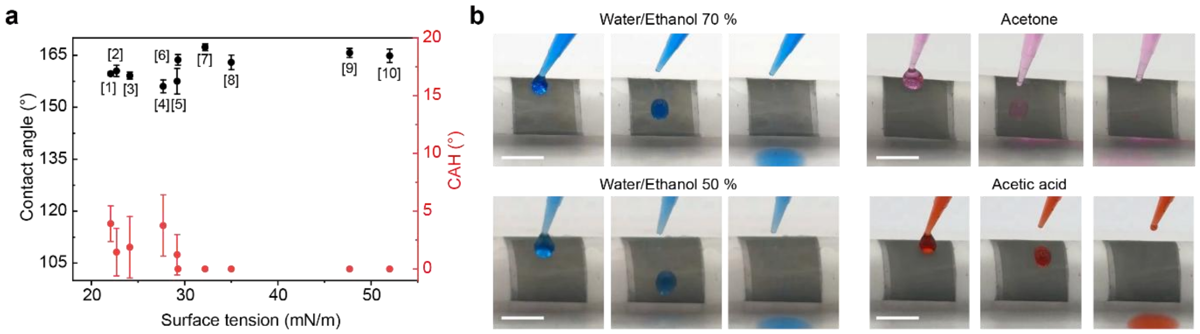

Figure S6. Organic liquid wettability on springtail mimetic superomniphobic surface (λ_{10}). (**a**) Contact angles and contact angle hysteresis (CAH) of organic liquid with varied surface tension ([1] Ethanol, [2] Acetone, [3] Water-ethanol 70 % mixture, [4] Acetic acid, [5] Water-ethanol 50 % mixture, [6] Acetonitrile, [7] Olive oil, [8] Water-ethanol 30 % mixture, [9] Ethylene glycol, and [10] Water-ethanol 10 % mixture). Data are presented as the mean ± standard deviation (n=3). (**b**) Photograph of liquid droplets rolling off from the curved superomniphobic surface. Scale bars: 1 cm.

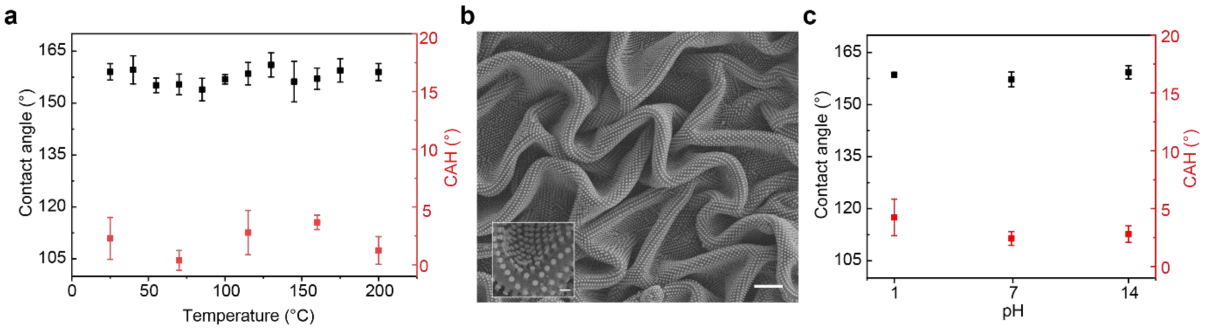

Figure S7. Stability tests of the curved superomniphobic surface. (**a**) Contact angle and contact angle hysteresis (CAH) of acetonitrile droplets on the surface exposed to temperature changes up to 200 °C. (**b**) SEM images of the surface after exposure at 200 °C. Scale bars: 10 μm allnd 1 μm. (**c**) Contact angle and CAH of acetonitrile droplets on the surface exposed to strong acid, salt, and alkali solutions. Data are presented as the mean ± standard deviation (n=3).

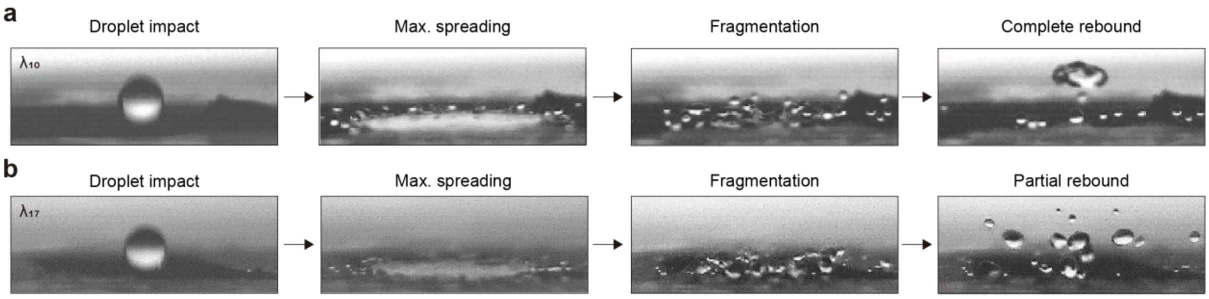

Figure S8. Droplet rebound dynamics on microwrinkle size-controlled surfaces. **(a, b)** Snapshot images of acetonitrile droplet rebound on **(a)** λ_{10} and **(b)** λ_{17} surfaces.

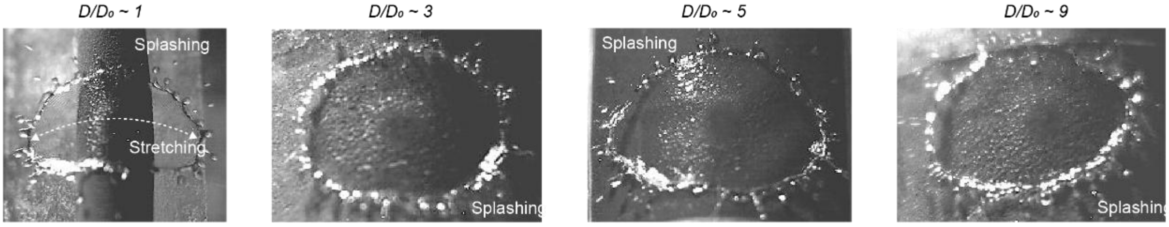

Figure S9. Snapshot images of the maximum spreading moment at *We* ~ 337.5 on each curved surface.

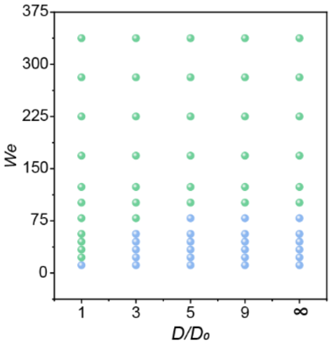

Figure S10. Converging break-up regime as a function of *We* and D/D_0 of the superomniphobic surface. Green colored regime indicates converging break-up and blue one indicates no break-up.

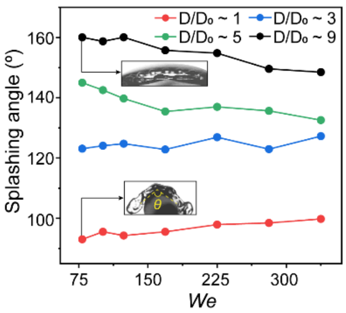

Figure S11. The splashing angle as a function of *We* on each curved surface.

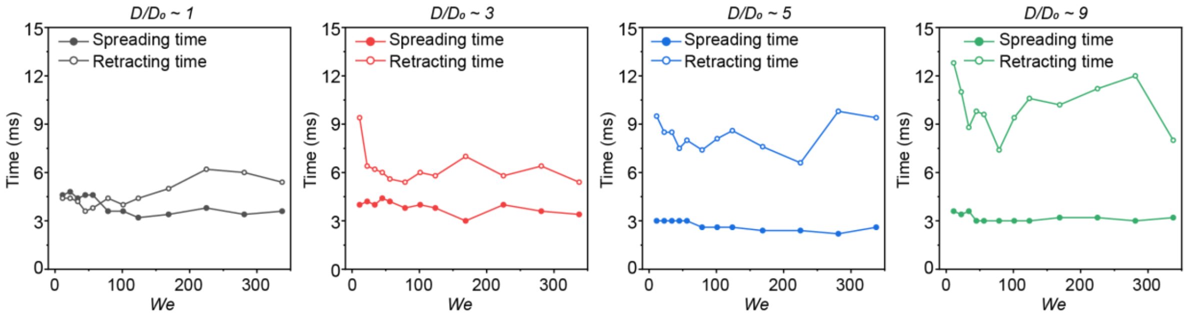

Figure S12. Spreading and retracting time on curved superomniphobic surfaces with different curvatures (D/D_0 ~ 1, 3, 5, and 9) as a function of *We*.

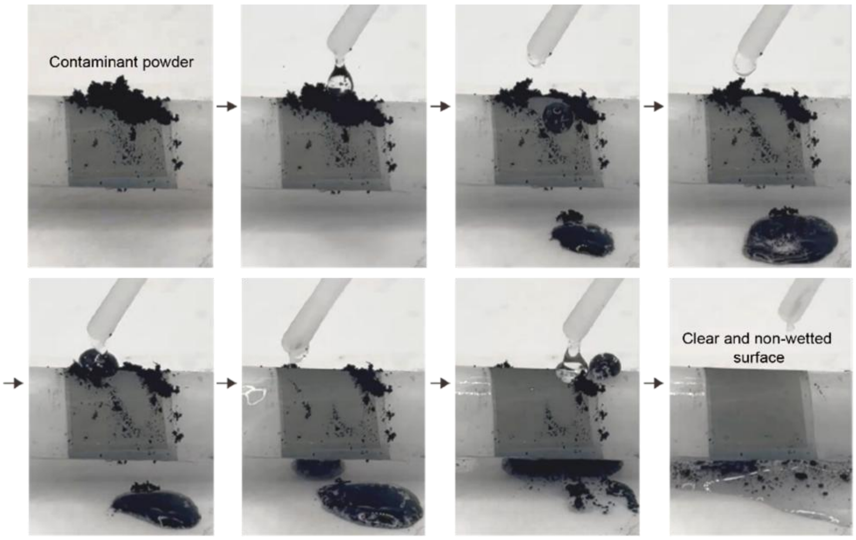

Figure S13. Photographs of the self-cleaning process on the curved superomniphobic surface with acetonitrile droplets.

Surface structure	Liquid	Surface tension (mN/s)	Droplet diameter (mm)	Impact velocity (m/s)	Contact angle (°)/ Contact angle hysteresis (°)	Contact time (ms)/ Contact time reduction (%)	Ref
Slippery liquid surface	Water	72	2.04	0.2-0.4	N.A.	10.2	[1]
Microbump structure	Water	72	3.4	1-1.5	N.A.	12-15/~5	[2]
Compact nanotexture	Water	72	2.3	1	160/1.6±0.5	18.8/14	[3]
PPFE coated micropillars	Water	72	2.15	1.31	157/10.6	23/11/26	[4]
Single ridge	Water	72	2.66	1.2	160/2	7.8/37	[5]
Curved hydrophobic rosettes coating	Water	72	2.9	0.63	160/3.2±0.6	11.6/40	[6]
NeverWet™ coated surface	Water	72	3.14	N.A.	160/15	13.7/25	[7]
Doubly reentrant structure	FC-72	10	1.02	0.1	160/N.A.	80/N.A	[8]
Dual scale re-entrant structure	20 wt% ethanol	32	N.A	N.A	151 ± 1/ 22 ± 1	18/N.A.	[9]
Springtail inspired structure	Ethanol	22.1	N.A.	1.17	150/N.A.	35.4/N.A.	[10]
Curved springtail inspired structure	Acetonitrile	29.29	2.14	1.47	160/~0	7.6/54	This work

Table S1. Comparative analysis of super-repellent surfaces reported in the literature and this work.

Liquid	Surface tension (mN/s)	Density (kg/m^3)	Viscosity (g/cm^3)	Droplet volume (uL)	Contact angle (º)	Contact angle hysteresis (º)
Ethanol	22.07	785	0.7850	6	159.67 ± 0.58	3.92 ± 1.55
Acetone	22.7	784	0.3600	6	160.48 ± 1.61	1.46 ± 2.06
Water-ethanol 70% mixture	24.12	863	0.8636	6	159.12 ± 1.00	1.86 ± 2.66
Acetic acid	27.7	1049	1.0560	6	156.03 ± 1.83	3.76 ± 2.66
Water-ethanol 50% mixture	29.2	910	0.9098	6	157.49 ± 3.69	1.24 ± 1.75
Acetonitrile	29.29	783	0.3430	6	163.67 ± 1.53	0
Olive oil	32.2	917	60.7000	6	167.34 ± 1.00	0
Water-ethanol 30% mixture	35	951	0.9506	6	162.96 ± 2.12	0
Ethylene glycol	47.7	1110	16.1000	6	165.68 ± 1.32	0
Water-ethanol 10% mixture	52	980	0.9971	6	164.85 ± 1.96	0

Table S2. Organic liquid wettability on the springtail-inspired superomniphobic surface.

References

[1] C. Hao, J. Li, Y. Liu, X. Zhou, Y. Liu, R. Liu, L. Che, W. Zhou, D. Sun, L. Li, L. Xu, Z. Wang, *Nat. Commun.* **2015**, *6*, 7986.

[2] S. Kim, Z. Wu, E. Esmaili, J. J. Dombroskie, S. Jung, *Proc. Natl. Acad. Sci.* **2020**, *117*, 13901.

[3] L. Wang, R. Wang, J. Wang, T.-S. Wong, *Sci. Adv.* **2020**, *6*, eabb2307.

[4] Y. Fan, C. Wu, J. Yang, Y. Wang, Y. Zhou, J. Zhou, J. Luo, J. Zhang, S. Huang, X. Tian, *Chem. Eng. J.* **2022**, *448*, 137638.

[5] J. C. Bird, R. Dhiman, H.-M. Kwon, K. K. Varanasi, *Nature* **2013**, *503*, 385.

[6] Y. Liu, M. Andrew, J. Li, J. M. Yeomans, Z. Wang, *Nat. Commun.* **2015**, *6*, 10034.

[7] X. Jiang, E. Xu, G. Wu, H. Z. Li, *Chem. Eng. Sci.* **2020**, *212*, 115351.

[8] T. “Leo” Liu, C.-J. “Cj” Kim, *Science* **2014**, *346*, 1096.

[9] H. Wang, Z. Zhang, Z. Wang, J. Zhao, Y. Liang, X. Li, L. Ren, *Chem. Eng. J.* **2020**, *394*, 124871.

[10] G.-T. Yun, Y. Kim, H. Ahn, M. Kim, G. M. Jang, S. G. Im, W.-B. Jung, H.-T. Jung, *ACS Nano* **2024**, *18*, 5622−5631.
